# Fate of free and bound phytol and tocopherols during fruit ripening of two *Capsicum* cultivars

**DOI:** 10.1038/s41598-020-74308-1

**Published:** 2020-10-14

**Authors:** Stephanie Krauß, Vanessa Hermann-Ene, Walter Vetter

**Affiliations:** Institute of Food Chemistry (170b), University of Hohenheim, Garbenstraße 28, 70599 Stuttgart, Germany

**Keywords:** Analytical chemistry, Mass spectrometry, Plant sciences, Natural variation in plants, Plant development

## Abstract

Phytol and tocopherols and their fatty acid esters (PFAE and TFAE) are isoprenoid lipid components which can be found for instance in vegetables. Their behavior during maturation of fruits and vegetables could reveal valuable information on their biosynthetic formation and biological function. As pods of the genus *Capsicum* contain considerable amounts of both PFAE and TFAE, two cultivars (i.e. *Capsicum annuum* var. *Forajido* and *Capsicum chinense* var. *Habanero*) were grown in a greenhouse project. The date of flowering and fruit formation of each blossom was noted and fruits were harvested in four specific periods which corresponded with different stages of ripening, i.e. unripe, semi-ripe, ripe and overripe. Quantification by means of gas chromatography mass spectrometry and creation of development profiles strongly supported the suggestion that PFAE and TFAE were formed as storage molecules during fruit ripening and parallel degradation of chlorophyll. Additionally, compound-specific carbon isotope ratios (δ^13^C values (‰)) of originally in PFAE and chlorophyll bound phytol ultimately proved that PFAE, besides tocopherols, serve as sink for the cytotoxic phytol moiety released from chlorophyll degradation during fruit ripening. Furthermore, color measurements were successfully implemented to simplify the usually cumbersome separation of chili fruits into different ripening degrees.

## Introduction

Both phytol and tocopherols are isoprenoid lipid components, which are widely spread in the plant kingdom^1,2^ Phytol (C_20_H_40_O) is mainly known as side chain of the green pigment chlorophyll and does hardly occur in its free form in plant tissue^3,4^. Free phytol has detergent-like properties which lead to a toxic impact on proteins and plant cell membranes^4^. In contrast, tocopherols (which exist in four different forms – α, β, γ and δ – depending on number and position of methyl substituents in the head group) serve as antioxidants and protect membrane lipids in plant cells from oxidative degeneration^1,5,6^. Hence, free tocopherols are found in photosynthetic tissues or in seeds whereas α-tocopherol (C_29_H_50_O_2_) usually represents the most abundant form followed by γ-tocopherol (C_28_H_48_O_2_)^6,7^. Besides, phytol and tocopherols can also be esterified to fatty acids, forming phytyl and tocopheryl fatty acid esters (PFAE and TFAE)^8–11^. Previous studies suggested that PFAE and TFAE could serve as storage forms for free phytol and tocopherols^3,4,12^. PFAE were mainly found to be produced in plants that suffer from stress like nitrogen deficiency, drought or during senescence^2,4,13^. As these conditions induce chlorophyll degradation which is accompanied by the release of free (cytotoxic) phytol, PFAE were assumed to be possible deactivating reagents^12,14,15^. Chlorophyll breakdown is also associated with fruit ripening, which can be observed for various fruits and vegetables by the change of color from green to red, orange, yellow or other shades^3^. Interestingly, PFAE were detected in ripe yellow and red but not in unripe green bell pepper fruits^12^. This indicated that the amount of PFAE in fruits and vegetables may increase during ripening. Although little knowledge exists so far, TFAE were thought to be formed as stable analogues when free tocopherols exceed a certain concentration^10^.

In this study we aimed to verify changes in the content and composition of PFAE and TFAE during ripening. Due to the high abundance of both PFAE and TFAE in fruits of the genus *Capsicum*^10,16^, we performed a greenhouse experiment with two *Capsicum* cultivars. Quantitative analyses by means of gas chromatography mass spectrometry (GC/MS) were supplemented with compound-specific stable isotope analysis (CSIA) of carbon isotopes (δ^13^C values (‰)) which allows conclusions on precursor molecules and underlying biosynthetic reactions^17,18^. Therefore, free phytol and tocopherols were separated from their esterified analogues by solid phase extraction (SPE) which enabled quantification and creation of developing profiles for individual compound classes with regard to ripening. Subsequent alkaline hydrolysis of the ester containing SPE fraction furthermore enabled the separate analysis of free and originally esterified phytol and tocopherols by gas chromatography coupled to stable isotope ratio mass spectrometry (GC-IRMS).

## Materials and methods

### Chemicals, consumables and standard materials

Solvents and chemicals used for lipid extraction, sample cleanup and derivatization as well as for synthesis of TFAE and PFAE standards were the same as reported before^10,12,16^. In addition, pyrogallol, acetic anhydride and acetic acid were purchased from Carl Roth (Karlsruhe, Germany). The carrier gas helium (quality 5.0) for GC/MS and GC-IRMS analysis as well as the working gas CO_2_ (quality 4.5, δ^13^C = – 30.5 ± 0.07‰) for GC-IRMS were from Westfalen (Münster, Germany) and the secondary reference material USGS40 (δ^13^C = – 26.4 ± 0.04‰ relative to Vienna Pee Dee Belemnite (VPDB)) was obtained from the Reston Stable Isotope Laboratory (Reston, VA). The α-tocotrienol standard was isolated from vitamin E capsules in our working group following the instructions of Vetter et al*.*^19^.

### Cultivation of *Capsicum* plants

*Capsicum* plants were cultivated under greenhouse conditions with the support of the *Service Unit Hohenheim Greenhouses* on campus of the University of Hohenheim, Stuttgart. Seeds of the *Capsicum* cultivars *Capsicum annuum* var. *Forajido* (*Jalapeño* type) (later on: “*Forajido*”) and *Capsicum chinense* var. *Habanero* (later on: “*Habanero”*) with guaranteed varietal purity according to European seed regulation were purchased from a local market-garden (Stuttgart, Germany) and planted mid November 2018 in cultivation soil. Juvenile plants were transferred into the greenhouse four weeks later in the middle of December. Temperature of the heating and ventilation system was set to 21 °C and 26 °C, respectively, during the days and 19 °C and 24 °C during the night. Cultivation substrate was on basis of sod peat with clay (pH 5.5 (CaCl_2_), salinity 1.5 g/L (KCl). Three months after sowing flowering began and 3–6 days later fruit formation occurred. Plants were fertilized twice per week starting four months after sowing and drip irrigation was carried out every 8 h for 15 min.

### Classification of chili fruits in four ripening stages

For determining different ripening stages, the date of flowering for each blossom was noted as was the date when fruit formation began. In the case of *Habanero* fruits, time from flowering to fruit formation was 4–5 days, for *Forajido* fruits this period was 3–6 days. The first ripening stage A was defined as “unripe” (Supporting Fig. S1) and fruits were harvested 28–29 (*Habanero*) or 42–44 (*Forajido*) days after beginning fruit formation. The date when color was changing from green to red (*Forajido*) or orange (*Habanero*) marked ripening stage B (semi-ripe) and was after ~ 38–45 and 48–55 days after fruit formation for *Habanero* and *Forajido*, respectively (Supporting Fig. S1). The next ripening stage C, defined as “ripe” was set at 7 (*Habanero*) and 4 (*Forajido*) days and the final ripening stage D (“overripe”) at 14 and 8 days after color change when the skin of the fruits started to appear wrinkled (Supporting Fig. S1). Fruits from four different plants of each cultivar were harvested, whereas two fruits per ripening degree were taken from each plant. Thus, for each ripening degree eight pods from four plants were analyzed.

### Color measurement as classifying criterion for ripeness of chili pods

After harvesting, color of the chili pods was measured immediately using a Spectrophotometer CM-700d with spherical geometry (Konica Minolta, Tokyo, Japan). Measuring parameters were as described before^20^ in the specular component excluded (SCE) mode with standard settings (2° observer, 3 mm aperture). Color was measured along an imaginary line with four measuring points from the stem to the tip of each chili pod from three sides and the mean value was calculated to yield the color coordinates a* (+ a* = red, −a* = green), b* (+ b* = yellow, −b* = blue) and L* (lightness: 0 = black, 100 = white) according to the Commission Internationale de l’Eclairage’s (CIE) L*a*b* color system for the whole fruit. After color measurement the samples were lyophilized, ground to gain a fine powder and stored at – 18 °C until further use. Water content was determined gravimetrically.

### Lipid extraction and sample clean up

Lipids were gained from ~ 1 g lyophilized sample by accelerated solvent extraction with ~ 80 mL of the solvent mixture cyclohexane/ethyl acetate (46:54, w/w). Operating parameters were as described by Weichbrodt et al*.*^21^ Solvent was removed by rotary evaporation and the residue was diluted in 4 mL of *n*-hexane. An aliquot of the lipid extract (1 mL) was used for solid phase extraction (SPE) which was carried out according to Krauß et al*.*^10^ Using this method, TFAE and PFAE (SPE fraction 2), free tocopherols and phytol (SPE fraction 3) and chlorophyll (SPE fraction 4) were eluted into three different fractions (Supporting Fig. S2). The volume of each fraction was adjusted to 1 mL *n*-hexane and aliquots corresponding to ~ 50 to 70 µg/mL were introduced to GC/MS analysis. SPE fraction 2 (TFAE and PFAE) was measured directly and SPE fraction 3 and 4 after silylation which was carried out according to Hammann et al*.*^22^.

### Identification and quantification by means of GC/MS

For identification and quantification purposes, TFAE and PFAE standards (esterified with the fatty acids 12:0, 14:0, 16:0, 18:0, 18:1*n*-9 and 18:2*n*-6) were prepared and purified following the instructions of Krauß et al*.*^10,12^ GC/MS analyses were performed in full scan mode (*m/z* 50–800) for identification as well as in selected ion monitoring (SIM) mode for quantification. The GC/MS setups used for the analysis of intact TFAE and PFAE (SPE fraction 2) as well as of free tocopherols and phytol (both trimethylsilyl (TMS) derivatives) were the same as described before.^10^ In short, intact TFAE/PFAE were analyzed on GC/MS system I which was equipped with a cool-on-column inlet and a Rtx-1 GC column (Restek, Bellefonte, PA). The GC/MS-SIM method included *m/z* 123.2, 143.2, 209.2, 217.2, 223.2, 237.2, 357.2, 474.5, 488.5, and 502.5 (dwell time was 35 ms each). Self-synthesized standards of α-TFAE-18:0, γ-TFAE-18:0 and PE-18:0 were combined to concentrations of 0.2 to 40 µg/mL, each. Free analogues were measured after silylation on GC/MS system II with a split/splitless injector operated in splitless mode and an HP-5MS GC column (Hewlett-Packard/Agilent, Waldbronn, Germany). Silylated tocopherols and phytol were determined by GC/MS-SIM via *m/z* 123.2, 143.2, 209.2, 217.2, 223.2, 237.2, 357.2, 474.5, 488.5, and 502.5 (dwell time 35 ms, each). Quantification was carried out by external calibration with a mixture of silylated standards of α-, β-, γ-tocopherol and phytol.

Limits of detection (LOD) were 0.05 and 0.02 µg/mL for PFAE and phytol, respectively, while limits of quantification (LOQ) were determined to 0.2 and 0.1 µg/mL. For α- and γ-tocopherol LOD/LOQ were 0.003/0.01 µg/mL and 0.002/0.01 µg/mL, respectively and for α-TFAE and γ-TFAE they lay at 0.08/0.3 µg/mL and 0.06/0.2 µg/mL.

Identification of both free and esterified tocopherols and phytol was carried out by comparison of their GC/MS spectra and retention times with standard solutions and literature data^10,11,23^.

### Saponification and acetylation

For CSIA, 500 µL-aliquots of SPE fractions 2 and 4 were separately saponified to release tocopherols and phytol bound either in fatty acid esters or in chlorophyll in accordance with methods described before^24–26^. In short, the samples were treated with 2 mL of a pyrogallol solution (60 g/L in ethanol), 1.8 mL of ethanol and 1.8 mL of aqueous potassium hydroxide (KOH) for 75 min at 80 °C after flushing the tube with nitrogen. After adding 3 mL of distilled water and 600 µL of acetic acid, the unsaponifiable matter was extracted with 3 mL of *n*-hexane. The *n*-hexane phase was washed twice with 1 mL of aqueous KOH solution (pH = 9). To avoid a dilution step during saponification, 2.4 mL of the *n*-hexane phase was transferred into a glass vial, and the final volume was adjusted to 400 µL. An aliquot of 200 µL of this solution was used for acetylation. After gently removing the solvent with nitrogen, each 100 µL of pyridine and acetic anhydride were added and the vial was heated to 60 °C for 1 h. Then, the derivatization reagents were removed and the residue was dissolved in 200 µL of *n*-hexane.

### Stable carbon isotope ratio analysis (δ^13^C values (‰)) of acetylated phytol and tocopherols by means of GC-IRMS

CSIA of carbon was performed on the same GC-IRMS setup with the same operating parameters as described before in the literature^20^. The injected sample volume was 1 µL. The GC oven program started at 55 °C and after 1 min temperature was increased first with 20 °C /min to 255 °C, then with 1.5 °C/min to 283 °C and finally with 15 °C/min to 325 °C which was held for 15 min. Helium was used as carrier gas with a column flow of 2.3 mL/min. All samples and standard solutions (mixture of acetylated phytol, α- and γ-tocopherol in concentrations of 10–50 ng/µL) were analyzed in triplicate and at the beginning and the end of each run, the working gas CO_2_ was measured five times. Furthermore, α-tocotrienyl acetate which was calibrated against USGS40 and used as internal working standard was added in the concentration of 20 ng/µL to each standard and sample solution. The optimal concentration range was determined to be 15–40 ng/µL for phytol and 20–40 ng/µL for both α- and γ-tocopherol. To prevent overloading the capacity of the oxidation reactor and enhance its lifetime, suitable time windows were chosen where the backflush valve that was installed prior to the oxidation reactor was activated to redirect high abundant analytes eluting from the GC column into waste. Standard deviations of δ^13^C values (‰) in sample solutions were 0.06–0.4‰ for phytol, 0.06–0.5‰ for α-tocopherol and 0.1–0.6‰ for γ-tocopherol. As the derivatization step altered the stable carbon isotopic composition of the analytes by adding two carbon atoms to the molecule with a different ^13^C/^12^C ratio, this influence was taken into account by carrying out a correction of the obtained δ^13^C values (‰) according to Eq. 1^27^ exemplarily shown for phytol.

$$\delta^{{{13}}} {\text{C}}_{{{\text{phytol}}}} (\textperthousand) \, = \, (({\text{n}}_{{{\text{phytyl}} - {\text{acetate}}}} \cdot \delta^{{{13}}} {\text{C}}_{{{\text{phytyl}} - {\text{acetate}}}} ) - ({\text{n}}_{{\text{acetyl group}}} \cdot \delta^{{{13}}} {\text{C}}_{{\text{acetic anhydride}}} ))/{\text{n}}_{{{\text{phytol}}}}$$

with n = number of carbon atoms of the particular compound.

## Results and discussion

### Simplified classification of chili fruits into different ripening stages by means of color measurement

The color of both *Capsicum* cultivars tremendously changed during ripening. This transformation of chloroplasts into chromoplasts is due to the formation and accumulation of hitherto concealed carotenoids^28,29^. Thus, the visual color impression of each cultivar is highly dependent on the individual and characteristic carotenoid profile and its changes. Accordingly, color changes could be used as criterion for the determination of ripening stages A to D although TFAE and PFAE are colorless. *Habanero* chili pods turned from green (stage A) over green-yellow (stage B) to orange (stages C, D), while *Forajido* fruits of stage B were green–red and turned full red in stage C and D. Accordingly, stage A represented unripe, stage B semi-ripe, stage C ripe and stage D overripe fruits. In the following, we aimed to connect these visual impressions with color measurements.

**Figure 1:**
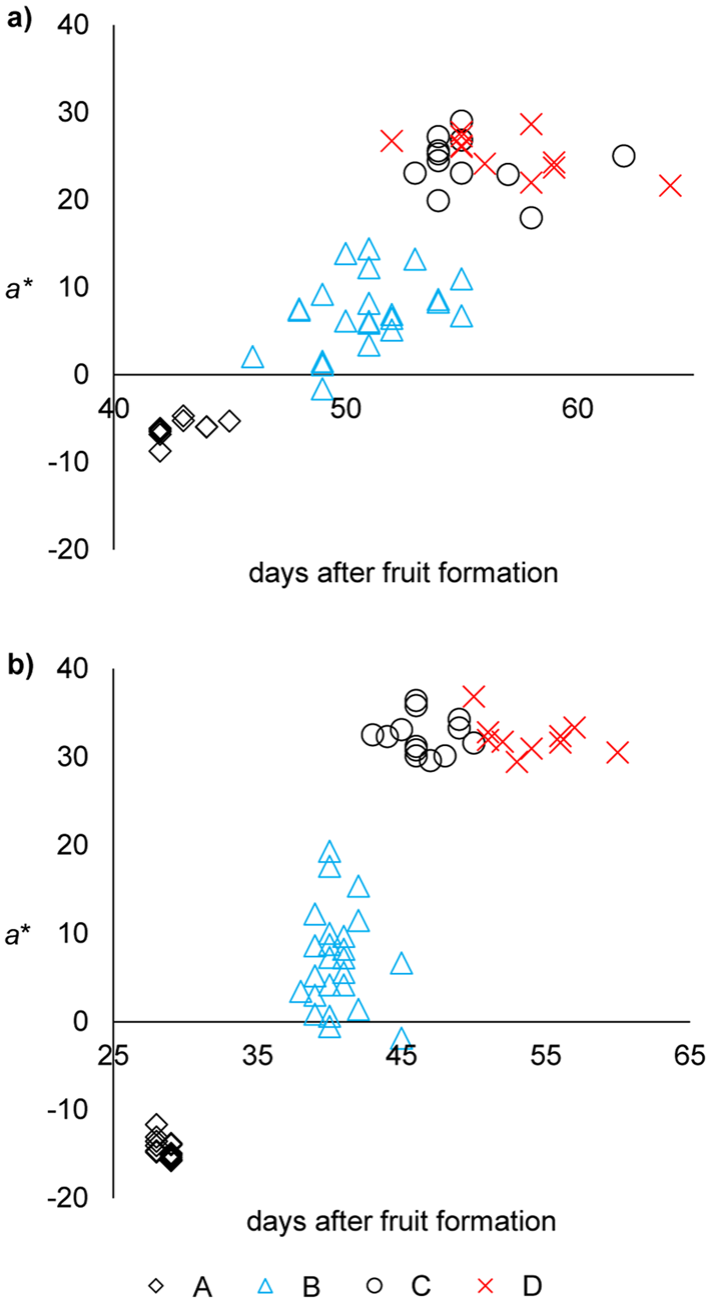
Scatter plot displaying the development of the color component a* (red-green) of **(a)** *Forajido* and **(b)** *Habanero* fruits in ripening stages A (unripe), B (semi-ripe), C (ripe) and D (overripe). The abscissa shows the number of days after fruit formation, when chili pods were harvested.

For both cultivars the change in color mainly affected the red-green component (a* value) which is negative with dominance of green and positive with dominance of red shares^30^ (Table 1). Accordingly, the color value a* showed the greatest change during ripening for both cultivars (Table 1) and most likely represents a function of occurring variations in the carotenoid profile. It turned out to be suitable for clearly separating ripe (stages C, D) from unripe (stage A) and semi-ripe (stage B) samples (Fig. 1). Raw data of a* including all samples were non-normally distributed (Shapiro–Wilk test, *p* > 0.05). Cluster-analysis (UPGMA) resulted in three groups: (i) samples of ripening stage A, (ii) semi-ripe pods of stage B and (iii) ripe fruits of stage C and D. Significance between these groups was verified by Kruskal–Wallis test (*p* < 0.05). Although a full differentiation between stages C and D via a* could not be achieved, our results demonstrate, that such color measurements can be used to classify chili pods with regard to ripening degrees. Plotting of a* over the number of days after fruit formation (which corresponds with the day of harvest after fruit formation), resulted in a full verification of stages A, B and C/D (Fig. 1). Internal cross validation of our data sets by means of single-case tests (*p* > 0.05) according to the leave-one-out method proved the excellent predictability of associating color values of chili pods with the distinct ripening stages “unripe”, “semi-ripe” and “ripe”. In future, color measurement based on a* is suggested for assigning samples to stages A, B and C/D which will substitute the cumbersome labeling of individual samples.

**Table 1:** Color values L* (lightness), a* (green–red), b* (blue-yellow) in the SCE mode of *Forajido* and *Habanero* fruits of different ripening degrees.

		Ripening degree			
		A (unripe)	B (semi-ripe)	C (ripe)	D (overripe)
		n^†^ = 10	n = 24	n = 12	n = 11
*Forajido *min–max (mean value)	L*	27.3 to 30.9 (28.9)	28.0 to 36.6 (31.2)	29.7 to 39.1 (33.7)	32.5 to 36.5 (35.1)
	a*	– 8.7 to – 4.7 (– 6.2)	– 1.6 to 14.4 (6.9)	18.0 to 29.0 (24.2)	21.6 to 28.7 (25.3)
	b*	6.0 to 9.8 (7.2)	5.2 to 13.2 (9.4)	9.7 to 15.5 (13.4)	12.0 to 15.0 (13.4)

		n = 13	n = 24	n = 13	n = 12
*Habanero* min–max (mean value)	L*	36.2 to 43.5 (39.6)	47.5 to 60.9 (54.2)	51.7 to 58.5 (54.3)	50.2 to 55.0 (52.4)
	a*	– 15.7 to – 11.7 (– 14.3)	– 1.9 to19.3 (7.0)	29.6 to 36.4 (32.4)	29.4 to 36.9 (31.9)
	b*	15.0 to 24.9 (20.2)	31.2 to 51.3 (41.6)	38.3 to 48.6 (41.8)	36.8 to 46.1 (40.6)

^†^n = number of samples.

### Natural variation in contents of free and esterified tocopherols and phytol in *Capsicum* plants

**Figure 2:**
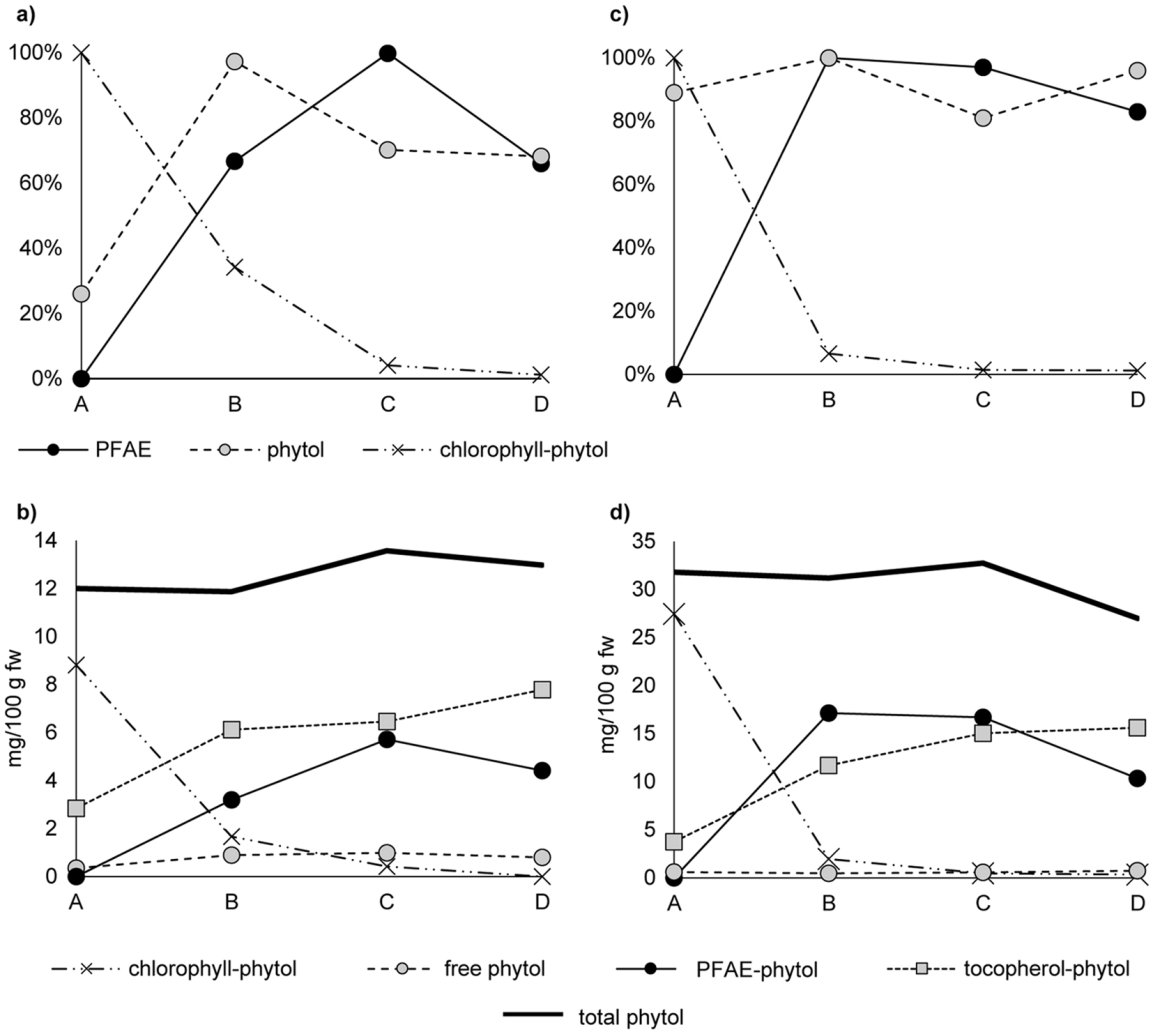
Increase and decrease of individual phytol compounds in % relative to the maximum content (= 100%) in **(a)** *Forajido* and **(c)** *Habanero* samples and absolute contents of phytol present in different components with their sum displayed as horizontal line in chili pods of ripening stages A (unripe), B (semi-ripe), C (ripe) and D (overripe) (**(b)** *Forajido*, **(d)** *Habanero*). The curves in **(a)** and **(c)** represent the mean of all samples of the four analyzed plants (standard deviation (SD) 8–18%). Contents shown in **(b)** and **(d)** are mean values of fruits of one plant (two pods per ripening degree per plant, mean deviation (MD) 0.03–0.3 mg/100 g fw).

The occurring natural variation in the contents of phytol, PFAE, tocopherols and TFAE within and between the plants was assessed by analysis of four plants of each cultivar. From each plant two fruits were taken per ripening degree which corresponds with eight fruits per plant and 32 fruits per cultivar. Considering the same ripening stage, minimum and maximum contents of each analyte were found to vary extensively from plant to plant (Table 2), although chili pods were harvested after the exact number of days after fruit formation was observed. Also, analyte contents in two fruits of the same ripening stage within one plant varied up to ~ 30% (data not shown). This observation was ascribed to influences like the extent of exposure of the individual fruits to sunlight due to the different position of the plants in the greenhouse and the different location of the single fruits on the plant stem. However, looking at each plant system individually, relative increasing or decreasing rates from one ripening stage to the next were relatively constant for both *Capsicum* cultivars. Hence, in the following, maximum values of all analytes were set to 100% and the corresponding values in the remaining ripening stages will be presented in percent of this value (Figs. 2a,c, 5a,c).

**Table 2:** Minimum and maximum contents (mg/100 g fw) of free and esterified tocopherols, free and esterified phytol and chlorophyll in fruits of the *Capsicum* cultivars *Forajido* and *Habanero* depending on ripening degree A-D.

	mg/100 g fw (n = 8 fruits per ripening degree and cultivar)								
	Ripening stage	PFAE	α-TFAE	γ-TFAE	free phytol	α-tocopherol	γ-tocopherol	β-tocopherol	chlorophyll^†^
*Capsicum annuum* var. *Forajido*	A	n.d	0.2–5.8	0.04–0.2	0.3–0.7	0.4–3.9	0.2–0.6	0.1–0.2	25.6–30.4
	B	4.9–18.0	1.4–8.6	0.1–0.4	0.8–4.5	3.0–9.4	0.3–0.7	0.1–0.3	5.0–15.8
	C	9.2–17.1	1.6–6.8	0.1–0.4	0.8–3.2	4.0–10.8	0.5–0.8	0.2–0.5	0.9–1.3
	D	7.5–18.9	1.5–8.5	0.1–0.5	0.8–2.8	7.3–11.8	0.4–1.5	0.4–0.7	n.d.– 0.7
*Capsicum chinense* var. *Habanero*	A	n.d	0.6–3.6	0.02–0.3	0.4–1.3	2.7–6.5	0.3–0.8	0.1–0.2	61.2–83.2
	B	6.6–17.9	1.5–18.2	0.1–0.6	0.1–1.8	6.7–17.1	0.1–0.8	0.1–0.3	3.7–13.7
	C	8.3–21.2	2.5–22.9	0.1–0.3	0.4–1.1	9.0–27.9	0.2–0.8	0.1–0.5	0.9–1.4
	D	5.9–11.7	3.7–32.2	0.04–0.3	0.3–2.6	8.2–27.1	0.3–0.7	0.1–0.4	n.d.– 1.5

^†^calculated from phytol content after saponification of chlorophyll-containing SPE-fraction by molar share of phytol in chlorophyll.

### Distribution of phytol in *Forajido* and *Habanero* chili pods in dependence of ripening degree

SPE was used to elute PFAE (SPE fraction 2), free phytol (SPE fraction 3) and chlorophyll-bound phytol into different fractions (SPE fraction 4). Saponification of SPE fraction 4 and subsequent silylation allowed us to quantify the share of phytol bound in chlorophyll (Supporting Fig. S2). Additionally, chlorophyll contents could be calculated since phytol makes up ~ 33% of its molecular mass^31^ (Table 2). This enabled us not only to track and compare the development of free phytol and PFAE during ripening but also of the vanishing of (phytol bound in) chlorophyll. For a more concise presentation, in the following, only results are discussed in detail for the cultivar *Forajido* followed by a short comparison with *Habanero* samples.

The highest chlorophyll-phytol level was detected in unripe fruits (stage A) followed by a steep decline in stage B (semi-ripe) and further in stage C (ripe), while chlorophyll was virtually absent in stage D (overripe) (Table 2, Fig. 2a). Free phytol strongly increased from stage A to stage B, but the amounts were generally low (< 3 mg/100 g fw, Table 2), so that its contribution to total phytol was small (Fig. 2b). By contrast, PFAE contents behaved strictly opposite to chlorophyll, which was verified by a Pearson correlation coefficient of ρ = – 0.9. Namely, PFAE were not detected in chili pods of stage A, but first appeared in stage B (Fig. 2a), where their contents instantaneously reached nearly their maximum level. In the ongoing ripening process (stages C, D), PFAE contents could further significantly increase (Kruskal–Wallis, *p* < 0.05) or stay at rather similar levels as in stage B (no significant changes, *p* > 0.05) (Fig. 2a). Noteworthy, total phytol composition seemed to vary only little between ripe fruits of stages C and D and changes were not significant (Fig. 2b; *p* > 0.05). Hence, variations in stages C and D were most likely due to natural variations within the fruits of one plant. However, we continued to treat both groups separately in order to have more independent data points for the evaluation of the different parameters. Nevertheless, the simultaneous increase of PFAE and decrease of chlorophyll supported the hypothesis that PFAE serve as deactivating storage form for high amounts of cytotoxic phytol released at once during chlorophyll degradation.

In addition to PFAE, chlorophyll-phytol could also be stored as tocopherols. Biochemically, tocopherol formation is closely linked with phytol. Namely, it starts with the conjunction of homogentisic acid and phytyl diphosphate^7,32,33^. Subsequent methylation and cyclisation reactions catalyzed by several enzymes result in γ-tocopherol which then serves as precursor for α-tocopherol^1,32^. Phytyl diphosphate stems either from de novo isoprenoid synthesis via the methylerythritol pathway in the plant chloroplasts or from degraded chlorophyll^1,33^. Previous studies showed that in many cases the tocopherol content increased significantly with the beginning of chlorophyll breakdown^1,33^, which indicated that released phytol could be directed into tocopherol synthesis. In α- and γ-tocopherol, the phytol moiety represents ~ 68% and ~ 71% of the molecular mass, respectively. Hence, to fully investigate the fate of degraded chlorophyll in our chili samples, in addition to the quantified amounts of free, chlorophyll- and PFAE-bound phytol, we also calculated the share of phytol bound in tocopherols and TFAE (in the following expressed as sum of both and defined as tocopherol-phytol) for each ripening stage and plant (Fig. 2b). Considering all major forms, the sum of phytol content was virtually the same in all ripening degrees (~ 12 mg/100 g fw). In stage B, C and D free phytol, chlorophyll- and PFAE-phytol alone did not sum up to the share of phytol originally bound in chlorophyll (9.2 mg/100 g fw) (Fig. 2b). For instance, in stage B, only 5.8 mg/100 g fw (63%) of chlorophyll-phytol of stage A (9.2 mg/100 g fw) was present as PFAE, free or still bound in chlorophyll. Yet, the remaining part of degraded chlorophyll (3.4 mg/100 g fw) was transferred into tocopherols, since total tocopherol-phytol from stage A (2.8 mg/100 g fw) increased to stage B by nearly the exact amount (3.3 mg/100 g fw) of chlorophyll-phytol that was not converted into PFAE or present as free phytol. Furthermore, from stage B to C, no considerable changes (Kruskal–Wallis, *p* > 0.05) could be observed in the share of tocopherol-phytol, but only in the amount of PFAE-phytol (*p* < 0.05), which indicated that the remaining chlorophyll-phytol was then primarily redirected into PFAE (Fig. 2b). Altogether, it was obvious that an almost quantitative conversion of chlorophyll-phytol into PFAE and tocopherols had occurred, and the free phytol assumedly represented the share of released phytol that was not yet transformed into non-toxic forms.

The observations made with *Forajido* (*Capsicum annuum*) samples were supported with *Habanero* (*Capsicum chinense*) plants but it happened even faster. Contrarily to *Forajido* pods, in *Habanero* samples PFAE already reached their maximum concentration in stage B and chlorophyll then was almost completely degraded (Fig. 2c). Again, the released phytol was directed into PFAE (~ 62%) and tocopherols (~ 38%) in stage B (Fig. 2d). This second example also indicates that slight variations in absolute phytol contents calculated from all major sources were due to natural variations, and that the sum of phytol present in the different compounds was the same for all ripening degrees. In conclusion, our results strongly suggest that, next to tocopherols, PFAE are a major sink for released phytol during chlorophyll breakdown in *Capsicum* plants and this seemed to be independent of species.

### δ^13^C values (‰) of phytol species in *Forajido* and *Habanero* chili pods in dependence of ripening stage and conclusions on their biochemical relationship

As biochemical reactions are associated with isotope fractionations, stable carbon isotope ratio analysis of phytol in its different forms could represent a possibility to further verify the transfer of chlorophyll-phytol into PFAE and tocopherols. For this purpose, δ^13^C values (‰) of phytol were measured in SPE fraction 3 (free phytol) and, after saponification, in SPE fraction 2 (PFAE) and SPE fraction 4 (chlorophyll). Since δ^13^C values (‰) differed slightly in individual plants (data not shown), each plant was considered as an individual system and only fruits from the same plant were compared to each other.

**Figure 3:**
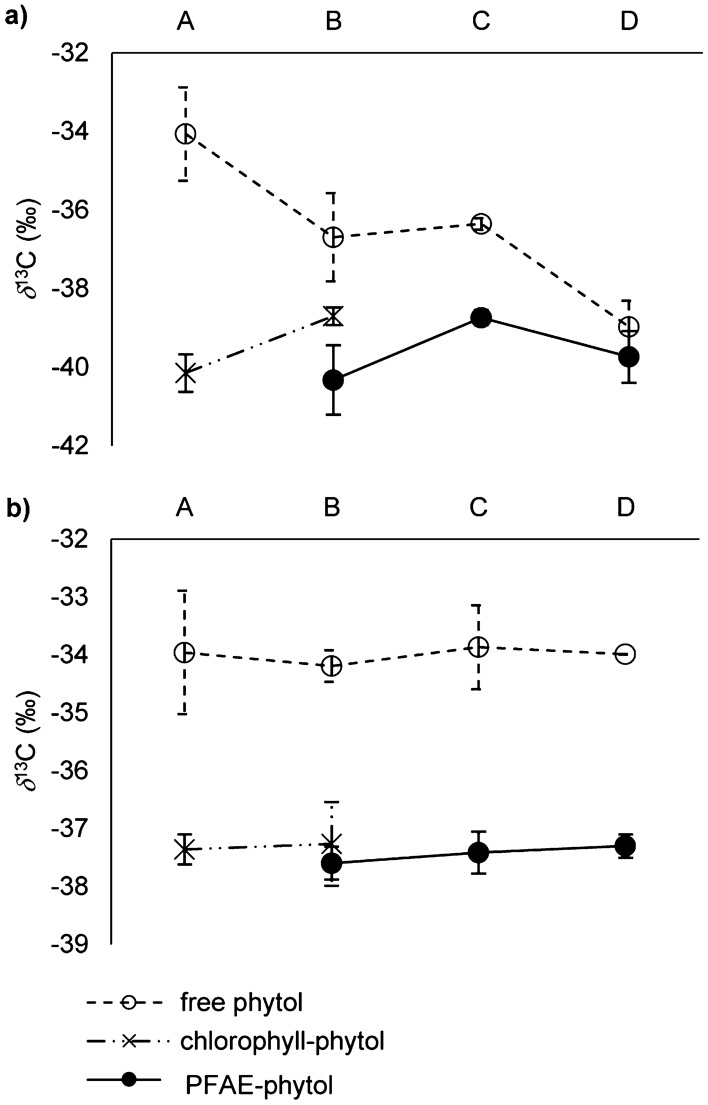
δ^13^C values (‰) (GC-IRMS) of chlorophyll-bound, free and PFAE-bound phytol in **(a)** *Forajido* and **(b)** *Habanero* chili pods of ripening stages A (unripe), B (semi-ripe), C (ripe) and D (overripe). Values represent means with standard deviation of the analysis of four fruits per ripening degree.

In *Forajido* plants, the δ^13^C value of chlorophyll-phytol increased from – 40.1‰ to – 38.7‰ from stage A to B (Fig. 3a). This enrichment in ^13^C of ~ 1.4‰ is due to chemical bonds being stronger between heavy isotopes (kinetic isotope effect). Hence, lighter isotopes (^12^C) tend to react faster, which results in an increase in ^13^C in the remaining chlorophyll-phytol in stage B (more positive δ^13^C values (‰)). In order to keep the balance (closed system^34^), the phytol released from chlorophyll must have been lighter in carbon than in stage A. From stage A to B, 81% of the initial chlorophyll pool was degraded and only 19% chlorophyll remained. This means, that the liberalized phytol should be (0.19 x – 1.4‰ = – 0.3‰) lighter in carbon than chlorophyll-phytol in stage A. According to Fig. 2b ~ 50% of the phytol released from chlorophyll was transferred into PFAE and the remaining share into tocopherols (section Distribution of phytol in *Forajido* and *Habanero* chili pods in dependence of ripening degree.). Conclusively, the δ^13^C value of phytol in PFAE should be (– 40.1‰ + (– 0.3‰ × 0.5) =) – 40.3‰. The recorded value of PFAE-phytol of – 40.3‰ was a perfect match of this calculation (Fig. 3a).

In the next step (stage B to C) the measured δ^13^C value (‰) of PFAE-phytol (– 40.3‰) increased by ~ 1.6‰ (to – 38.7‰). According to our results from above, from stage B (100%) to stage C chlorophyll declined by further ~ 70% and none of this additionally released phytol was transformed into tocopherols (Fig. 2b), but exclusively into PFAE. Consequently, the δ^13^C value (‰) of the remaining chlorophyll-phytol in stage C would be expected to be by (0.25 x – 1.6‰ =) – 0.4‰ lighter in carbon than of stage B, resulting in a δ^13^C value of – 38.7‰ + (– 0.4‰) = – 39.1‰. The initial enrichment (stage A to B) and subsequent depletion (stage B to C) in carbon of remaining chlorophyll is in full agreement with the kinetic isotope effect mentioned above. Due to the observation that chlorophyll-phytol after stage B was almost completely transformed into PFAE and the absence of chlorophyll in stage D, the δ^13^C value (‰) of PFAE-phytol (stage D) should approximate the original value of chlorophyll-phytol in stage A (δ^13^C = – 40.1‰) which was also observed within small margins (Fig. 3a). Slight variations between calculated and measured δ^13^C values (‰) could be ascribed to either uncertainties of the measurements or further redirection of released phytol into tocopherol synthesis (see below). In addition, free phytol was by ~ 3–5‰ more enriched in ^13^C than PFAE and chlorophyll (Fig. 3a). Again, partial transfer of free phytol into tocopherols will be realized by utilization of isotopically light phytol and the remaining share would get enriched in ^13^C.

In comparison to *Forajido* samples, in *Habanero* fruits the conversion of chlorophyll-phytol into PFAE and tocopherols was nearly completed (~ 93%) in stage B (Fig. 2c,d). This explained the very small differences between δ^13^C values (‰) of chlorophyll-phytol in stage A (– 37.4‰) and stage B (– 37.2‰) on the one hand and between chlorophyll-phytol (stage A) and PFAE-phytol (– 37.5 to – 37.3‰) on the other hand (Fig. 3b). Furthermore, δ^13^C values (‰) of free phytol were also by 3–4‰ more positive than those of chlorophyll- or PFAE-phytol and hence, supported our results obtained for *Forajido* samples (Fig. 3b). The very good match of predicted and measured δ^13^C values (‰) and the confirmation of reported trends and dimensions in both chili cultivars ultimately prove that PFAE were the predominant sink of phytol released from chlorophyll.

### Fatty acid pattern of PFAE and changes during ripening

For both *Capsicum* cultivars, phytol was mostly esterified with saturated and longer chained fatty acids (16 to 25 carbon atoms), which was in accordance with previous results in other chili pepper pods (unknown cultivars)^10^, but also shorter chained fatty acids (C_10_ – C_14_) were detected. PFAE were reported to be part of the wax ester fraction of plant leaves^8,13,23,35^, which usually comprise mainly long chain fatty acids and alcohols. This could explain the preferential esterification of these fatty acids with phytol. Nonetheless, one peak eluting shortly prior to PFAE-18:0 featured 18:1*n*-9 (shown after saponification because 18:1*n*-9 and 18:2*n*-6 co-elute on the nonpolar GC column of GC/MS system I and M^+^ could not be detected). Additionally, two very low abundant peaks detected in some samples possibly represented the unsaturated PFAE of 20:1 and 22:1, which could not be fully verified due to their extremely low amounts.

**Figure 4:**
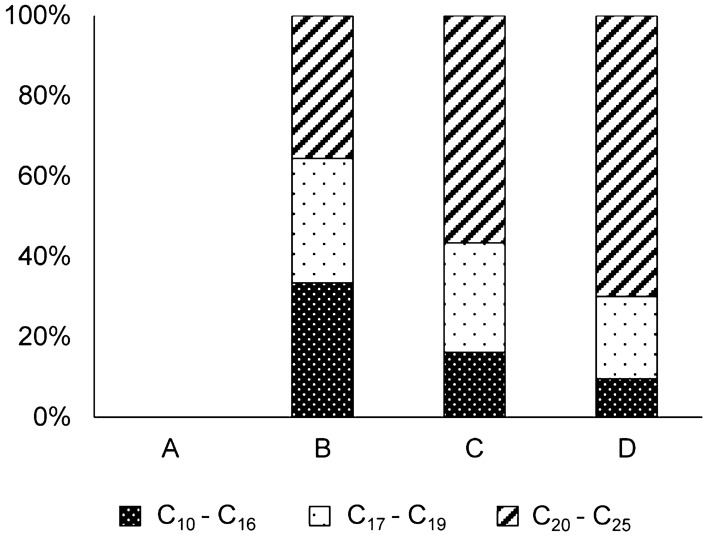
Fatty acid composition (in groups of chain length) of PFAE in *Forajido* and *Habanero* chili pods of ripening stage B (semi-ripe), C (ripe) and D (overripe). Note, that no PFAE were detected in stage A (unripe) and that monoenoic fatty acids are included in the calculated shares of each group.

Interestingly, PFAE showed remarkable changes in the fatty acid patterns depending on ripening degree of the fruits (Fig. 4). Namely, the share of short and medium chain fatty acids (< C_17_) decreased considerably from stage B (where PFAE were first detected) to stage C and finally stage D in favor of longer chained fatty acids (> C_20_) (Fig. 4). One explanation for this observation could lie in the availability of fatty acids. In early ripening stages bigger amounts of short and medium chain fatty acids might be available that could serve temporarily as deactivating reagent for instantaneously released phytol. In the maturation of fruits, however, the share of long chain fatty acids available for esterification probably increases and hence, enables the storage of phytol in highly nonpolar lipid components like wax esters. Another explanation could be that chlorophyll degradation might start in the inner parts where more short/medium chain fatty acids are available and finally on the external wax layer where long chain fatty acids are preferably utilized. Unfortunately, this effect could not be studied by IRMS yet, since CSIA of the intact PFAE was not possible so far due to co-elutions of PFAE and TFAE on the GC column.

### Distribution of free and esterified tocopherols in dependence of ripening degree

Both *Forajido* and *Habanero* fruits contained free α-, β- and γ-tocopherol as well as their fatty acid esters. Fatty acids were mainly bound to α-tocopherol (> 90%)^10^. β- and γ-TFAE could not be differentiated due to co-elution, but saponification of the ester fraction verified a γ-/β-tocopherol ratio of 19, which corresponds with < 9.5% γ-TFAE and < 0.5% β-TFAE.

**Figure 5:**
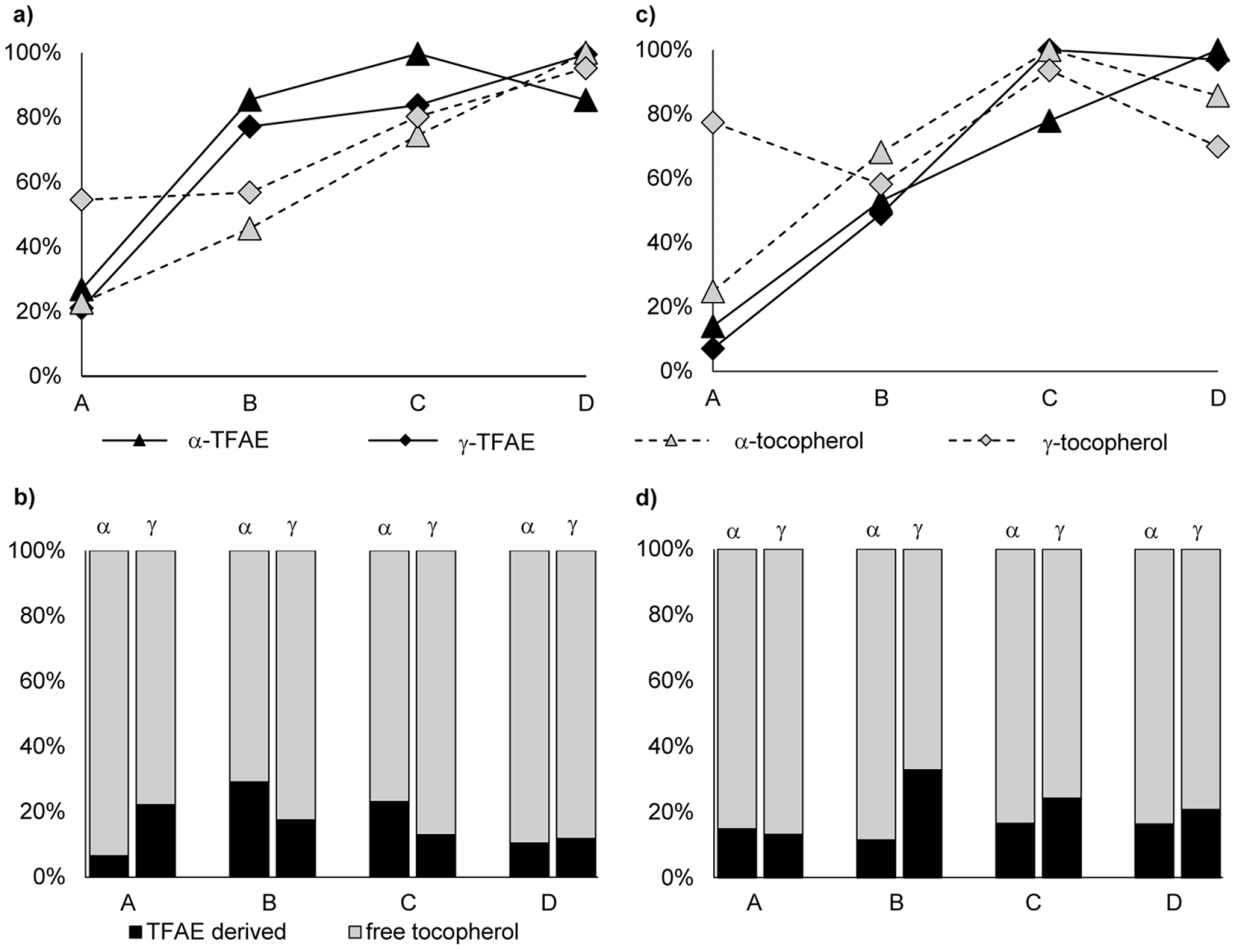
Increase and decrease of free and esterified tocopherols in % relative to the maximum content (= 100%) in **(a)** *Forajido*, **(c)** *Habanero* samples and percentage distribution of free and esterified α- and γ-tocopherols (**(b)** *Forajido*, **(d)** *Habanero*) in chilies of ripening stage A (unripe), B (semi-ripe), C (ripe) and D (overripe). The curves and bars represent the mean values of all samples of the four analyzed plants (SD 2–15%).

Similarly to phytol species, absolute contents of free and esterified tocopherols varied extensively within plants (Table 2). Hence, the investigation of potential changes during ripening was carried out after normalizing the contents of each component relative to its maximum concentration (100%) (Fig. 5). In contrast to PFAE, TFAE were already present in fruits of stage A (Fig. 5a,c), although their initial share of total tocopherol was < 20% for both *Forajido* and *Habanero* (Fig. 5b,d). In the further ripening process, concentrations of both free and esterified tocopherols increased significantly (Kruskal–Wallis, *p* < 0.05) (Fig. 5a,c). This verified the assumption that TFAE are formed as stable storage molecules for free tocopherols, when these exceed a certain concentration. Noteworthy, in *Forajido* samples TFAE reached already ~ 85% of their maximum concentration in stage B (changes to stages C/D were statistically non-significant, *p* > 0.05), while in *Habanero* fruits another steep, significant (*p* < 0.05) increase of TFAE from stage B (~ 50%) to stage C (~ 80% for α-TFAE, 100% for γ-TFAE) could be observed (Fig. 5a,c). This might be due to the considerably higher free (especially α-) tocopherol contents in *Habanero* chilies, which were in stage B partly already twice as high as those in *Forajido* samples (Table 2). Although differences between the increasing rates of TFAE and free tocopherols affected the total tocopherol composition – especially in fruits of stage B – the contribution of TFAE to total tocopherol stayed < 30% for both cultivars (Fig. 5b,d).

### Fatty acid pattern of TFAE and changes during ripening

**Figure 6:**
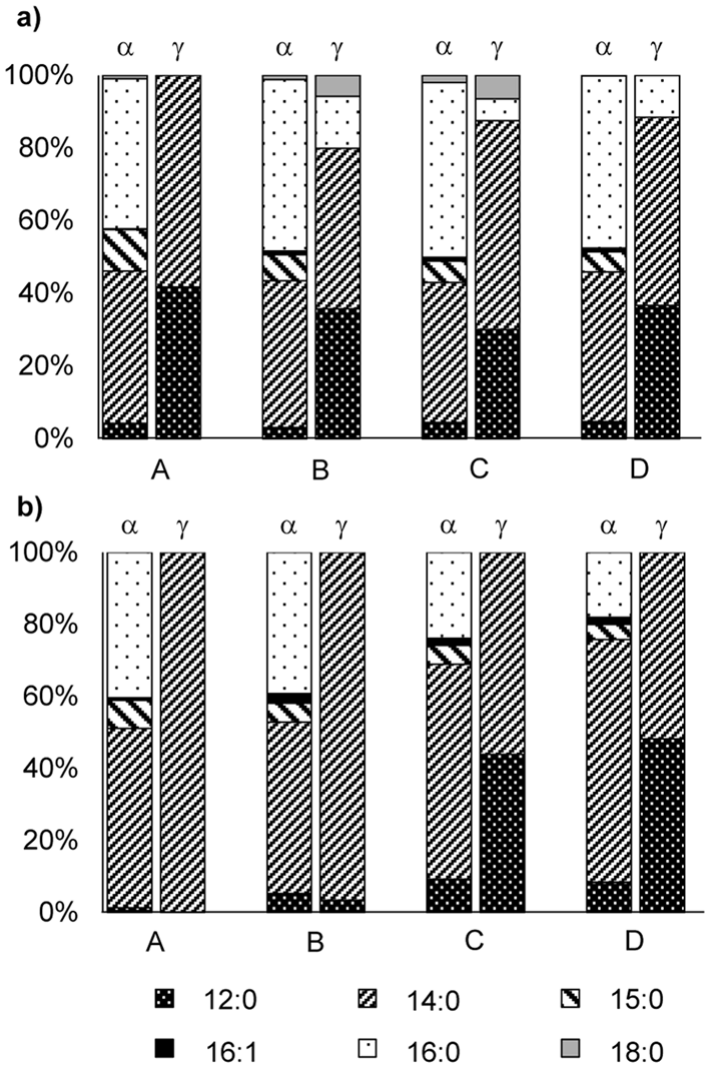
Fatty acid pattern of α- and γ-TFAE of **a)** *Forajido* and **b)** *Habanero* chili pods of ripening stages A (unripe), B (semi-ripe), C (ripe) and D (overripe). Values are means of all analyzed fruits.

Similarly to bell pepper^10^, α- and γ-tocopherol were mainly esterified with the fatty acids 12:0, 14:0, and 16:0. In addition, lower amounts of 18:0 were detected in *Forajido* samples and also α-tocopherol esters of 15:0 and 16:1*n*-7. However, it was remarkable that 14:0 and 16:0 were predominantly α-TFAE and 12:0 and 14:0 dominated in γ-TFAE (sum at least 80% for both groups) (Fig. 6). This was observed in all fruits and all ripening stages of both cultivars and produced evidence that α-TFAE were predominantly formed after the transfer of γ- into α-tocopherol. Similarly to PFAE, TFAE were detected in the wax ester fraction of plants^9,36^, which preferably comprise long chained fatty acids. This was in full agreement with our conclusion that γ-tocopherol must have initially been converted into α-tocopherol followed by the esterification with the longer chained fatty acids 14:0 to 18:0 before the remaining short chained fatty acids were esterified with α- and γ-tocopherol. Interestingly, while in *Forajido* chilies the share of α-TE-16:0 varied only slightly (41–48%) during ripening (Fig. 6a), in *Habanero* samples it decreased from 40% (stage A) to 18% in favor of mainly α-TE-14:0 (stage D) (Fig. 6b). In *Forajido* samples the TFAE content stayed at more or less the same level from stage B to D (Fig. 5a), which explains the consistency in their fatty acid pattern. By contrast, the amount (%) of TFAE increased continuously from stage B to D in *Habanero* fruits (Fig. 5c). The changing fatty acid pattern in TFAE towards higher shares of 12:0 and 14:0 indicated, that the high concentrations of free tocopherols and thus high demand for TFAE as storage molecules required the incorporation of also shorter chained fatty acids in addition to the preferred longer chained ones (16:0, 18:0).

### δ^13^C values (‰) of free and esterified tocopherols in *Forajido* chili pods

Similarly to phytol, saponification of the TFAE containing SPE fraction (SPE fraction 2) allowed us to compare the stable carbon isotope composition of free tocopherols (SPE fraction 3) and originally esterified tocopherols (SPE fraction 2) by GC-IRMS (Supporting Fig. S2).

**Figure 7:**
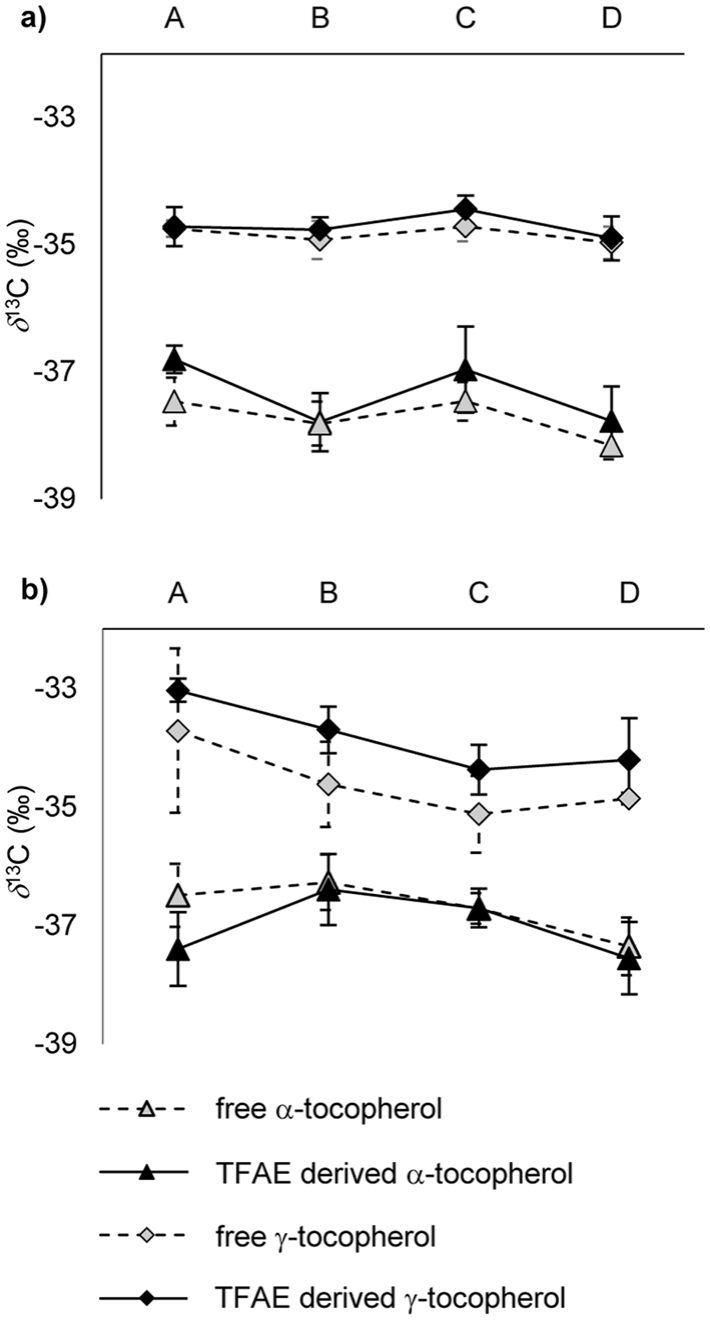
δ^13^C values (‰) (GC-IRMS) of free and esterified α- and γ-tocopherol in **(a)** *Forajido* and **(b)** *Habanero* chili pods of ripening stages A (unripe), B (semi-ripe), C (ripe) and D (overripe). Values represent means with standard deviation of the analysis of four fruits per ripening degree.

In *Forajido*, δ^13^C values (‰) of α-tocopherol (and TFAE derived α-tocopherol) generally were by ~ 4‰ and thus significantly (Kruskal–Wallis, *p* < 0.05) more negative than of γ-tocopherol (and TFAE derived γ-tocopherol) (Fig. 7a). This difference was most likely caused by the kinetic isotope effect associated with enzymatic conversion of γ- into α-tocopherol. The ratio of α- to γ-tocopherol was ~ 9:1 irrespective of the ripening stage. Hence, in stage A, 90% of γ-tocopherol was already transferred into α-tocopherol (δ^13^C = – 37.5‰) which resulted in a δ^13^C value of – 34.8‰. By adoption of this relationship, the original δ^13^C value (‰) of γ-tocopherol should have been (0.1 x – 34.8‰ + 0.9 x – 37.5‰ =) – 37.2‰. This relationship, which was fully confirmed with δ^13^C values (‰) obtained for α- and γ-tocopherol for *Habanero* fruits (Fig. 7b), verified our conclusion from above. Namely, isotopically light phytol released during chlorophyll-breakdown must have been directed into tocopherol synthesis. In order to exactly determine the contribution of chlorophyll-phytol to the δ^13^C value (‰) of tocopherols, it would be necessary to know the stable isotope composition of the second key element of tocopherol synthesis, i.e. homogentisic acid. However, our results show, that even without that knowledge, the biochemical relationship between chlorophyll-phytol and tocopherols could be clearly demonstrated by connecting their δ^13^C values (‰) with existing ratios between individual tocopherols and between tocopherols and chlorophyll-phytol.

TFAE derived α- and γ-tocopherol had each very similar (Kruskal–Wallis, *p* > 0.05) δ^13^C values (‰) to their free analogues (Fig. 7a). This suggested that the kinetic isotope effect associated with the esterification of tocopherols with fatty acids was rather small in comparison to the transfer of γ- into α-tocopherol. Furthermore, variations in δ^13^C values (‰) of free or originally esterified tocopherols lay within small margins between fruits of different ripening stages (Fig. 7a) and were statistically non-significant (*p* > 0.05). This was in accordance with the small changes occurring in the composition of total tocopherols during maturation (Fig. 5b). Results were similar for *Habanero* chili pods, although differences between δ^13^C values (‰) of tocopherols in different ripening stages were slightly bigger (Fig. 7b). Hence, δ^13^C values (‰) of tocopherols for different ripening stages not only verified the results obtained from quantitative analysis but also supported the conclusions from above regarding their biochemical relationship with chlorophyll-phytol.

### Connecting color measurements with TFAE content

**Figure 8:**
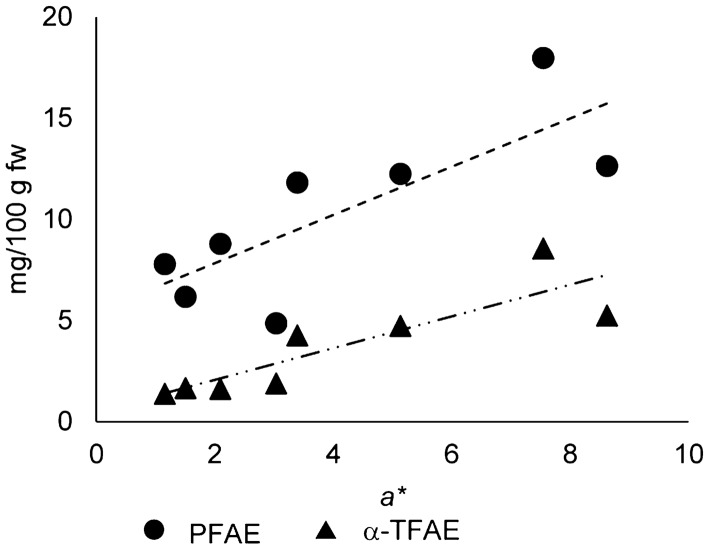
Scatter plot of α-TFAE and PFAE-content (mg/100 g fw) against the color component a* (red-green) for *Forajido* fruits of the ripening degree B (semi-ripe). The dotted lines indicate existing correlations with a Pearson correlation coefficient of ρ ≈ 0.9 for α-TFAE and ρ ≈ 0.8 for PFAE.

Interestingly, a* values of *Forajido* samples of ripening stage B correlated well (ρ = 0.86, Pearson correlation test) with α-TFAE content (Fig. 8). Hence, color values may not only allow to draw conclusions on carotenoid contents and profiles as mentioned above. In addition, this parameter could also serve as a marker for varying TFAE concentrations. Namely, fruits of ripening degree B with lowest a* values also had the lowest α-TFAE contents, while high a* values corresponded to high amounts of esterified α-tocopherol. This produced strong evidence that α-TFAE were newly formed during ripening and supported our conclusions from above. For PFAE, correlation was not as strong as for α-TFAE but still existed with a Pearson correlation coefficient of ρ = 0.78 (Fig. 8). Based on these findings, we suggest, that measurement of a* could not only replace the cumbersome labeling of fruits for assignment of ripening degree but might additionally indicate the order of magnitude of α-TFAE and PFAE content of *Forajido* fruits of stage B without elaborate extraction, purification and quantification procedures.

## Conclusions

The separate quantitative analysis of free and esterified tocopherols and phytol as well as chlorophyll-phytol in chili pods of the *Capsicum* cultivars *Forajido* (*Capsicum annuum*) and *Habanero* (*Capsicum chinense*) of different ripening stages allowed us to study the development of each compound class during fruit maturation. The color value a* was found to be suitable for classifying chilies into unripe (stage A), semi-ripe (stage B) and ripe (stage C/D) fruits and in the future could substitute the cumbersome labeling of individual samples for determining the ripening degree. Since the naturally occurring variation of the different compounds between the plants was quite extensive, it was important to investigate each fruit and plant individually. Calculation of the phytol contents bound in chlorophyll, PFAE and tocopherols in each ripening stage proved that besides tocopherols, PFAE were the predominant sink for phytol released during chlorophyll-breakdown. This finding was further supported by CSIA (δ^13^C values (‰)) of free and esterified phytol and tocopherols. Changes in their δ^13^C values (‰) from one ripening stage to the next could be fully explained by calculations based on observed changes in ratios of their contents and compositions. Reported trends and dimensions were similar for both *Capsicum* cultivars, suggesting that the observed mechanisms for deactivating cytotoxic phytol released from chlorophyll degradation are the same within the genus *Capsicum*, irrespective of species.

## Supplementary information

Supplementary file1

Supplementary file2

## Data Availability

All data relevant to this research are included in the article or provided as supplementary material.
