# Supplementary figures and images for: Fate of free and bound phytol and tocopherols during fruit ripening of two Capsicum cultivars
Source: Sci Rep. 2020 Oct 14;10:17310. doi: 10.1038/s41598-020-74308-1 (PMC7560742; doi:10.1038/s41598-020-74308-1)

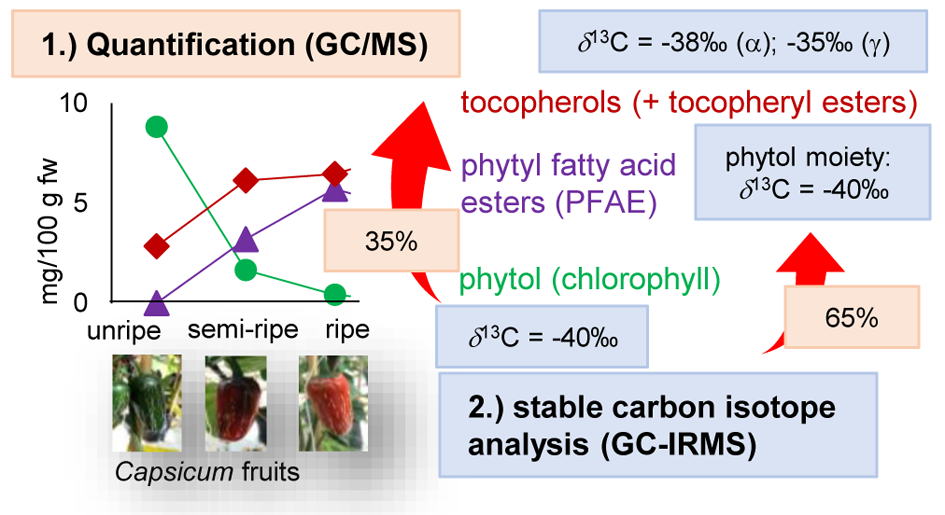

Supplement: Supplementary file 1 — Supplementary file1 [file 41598_2020_74308_MOESM1_ESM.tif]
