# Supplementary material for: Fate of free and bound phytol and tocopherols during fruit ripening of two Capsicum cultivars
Source: Sci Rep. 2020 Oct 14;10:17310. doi: 10.1038/s41598-020-74308-1 (PMC7560742; doi:10.1038/s41598-020-74308-1)
Supplement: Supplementary file 2 — Supplementary file2 [file 41598_2020_74308_MOESM2_ESM.docx]

**Supporting information for the manuscript:**

**Fate of free and bound phytol and tocopherols during fruit ripening of two *Capsicum* cultivars**

Stephanie Krauß, Vanessa Hermann-Ene, Walter Vetter*

University of Hohenheim, Institute of Food Chemistry (170b), Garbenstraße 28, 70599 Stuttgart, Germany

*Corresponding author

Walter Vetter

Phone: +49 711 459 24016

Fax: +49 711 459 24377

e-mail: [walter.vetter@uni-hohenheim.de](mailto:walter.vetter@uni-hohenheim.de)

**Fig. S1:** Pictures of *Forajido* (left) and *Habanero* (right) chili pods of different ripening stages: unripe (A), semi-ripe (B), ripe (C) and overripe (D).

**Fig. S2:** Flow chart for the separation of free and esterified phytol/tocopherols and chlorophyll by solid phase extraction (SPE) with subsequent saponification of the TFAE/PFAE- and chlorophyll-containing SPE fractions to quantify their phytol/tocopherol shares.
